# Two-Decade Retrospective Analysis of Endogenous Endophthalmitis in Spain and Mexico: A Comprehensive Study

**DOI:** 10.3390/jcm13174990

**Published:** 2024-08-23

**Authors:** Elia de Esteban Maciñeira, Manuel F. Bande, Jorge Ivan Soberanes-Pérez, Laura Paniagua, Maria F. Golzarri, Jans Fromow-Guerra, María José Blanco Teijeiro, Rosario Touriño Peralba

**Affiliations:** 1 Servicio de Oftalmología, Complejo Hospitalario Universitario de Santiago de Compostela, 15706 Santiago de Compostela, Spain; elia.de.esteban.macineira@sergas.es (E.d.E.M.); maria.jose.blanco.teijeiro@sergas.es (M.J.B.T.); rosario.tourino@usc.es (R.T.P.); 2 Unidad de Retina, Asociación para Evitar la Ceguera en México, Ciudad de Mexico 04030, Mexico; drsoberanesperez@gmail.com (J.I.S.-P.); maria.golzarri@apec.com.mx (M.F.G.); fromow@me.com (J.F.-G.); 3 Servicio de Oftalmología, Complejo Hospitalario Universitario de Ferrol, 15405 Ferrol, Spain; laurapf4@hotmail.com

**Keywords:** endophthalmitis, microbial patterns, antibiotic resistance, risk factors

## Abstract

**Objectives:** The aim of this study was to investigate endogenous endophthalmitis (EE) in Spain and Mexico, focusing on microbial patterns, antibiotic resistance, infection sources, risk factors, and patient outcomes. **Methods:** Over 20 years, 705 endophthalmitis cases were reviewed, and we identified 78 cases of EE in Santiago de Compostela, Spain, and Mexico City, Mexico. Microbial etiology, infection sources, antibiotic resistance, and treatment outcomes were compared between patients from Spain and Mexico. **Results:** Among the 78 EE cases, 47 (60.25%) were from Spain and primarily had bacterial infections (57.1%, mainly Staphylococcus and Streptococcus). In contrast, 31 cases (39.74%) were from Mexico and had a higher prevalence of fungal infections, particularly Candida (47.1%). Diabetes mellitus was a significant risk factor, and was more common in Mexico (61.3%) than in Spain (37.0%). The Spanish cohort exhibited notable antibiotic resistance, especially in Staphylococcus. Treatment typically involved systemic and intraocular antibiotics, with vitrectomy performed in 61.5% cases. Post-treatment, bacterial infections had higher success rates (approximately 50%) compared with fungal infections (approximately 30%). Evisceration was necessary in 9% cases, and the overall mortality rate was approximately 4.4%; it was slightly higher in Mexico than in Spain. **Conclusions:** The study highlights significant regional differences in EE between Spain and Mexico, particularly regarding microbial etiology and antibiotic resistance. The findings emphasize the need to adapt healthcare practices to specific regions to improve EE treatment outcomes, underscoring the importance of ongoing research and interregional collaboration to better understand and manage this complex condition.

## 1. Introduction

Endogenous endophthalmitis (EE) is a rare intraocular infection that can be visually devastating. EE accounts for approximately 2–8% of all endophthalmitis cases [1]. Prompt diagnosis and treatment are crucial for optimal visual outcomes; however, the origin of the underlying infection remains unidentified in several cases.

EE can be caused by both bacteria and fungi, with approximately half of the reported cases being of bacterial origin. The main risk factors for EE include an immunocompromised status (such as chronic use of corticosteroids, malignancy, end-stage liver/kidney disease, organ transplantation, diabetes mellitus), intravenous drug use, intravenous catheters, or distant abscesses. In rare cases, EE has also been reported in healthy individuals [2,3,4].

The infectious agent travels through the bloodstream and multiplies in the choroid, eventually infiltrating the retina and extending into the vitreous humor. Patient presentations can range from asymptomatic to symptoms typical of severe uveitis, including painful red eyes with reduced vision. Although EE is more often unilateral, up to one-third of the cases may have bilateral involvement [5,6].

This multicenter observational study aimed to address EE by comparing cohorts in Spain and Mexico. Our results will provide valuable insights into the demographic, etiological, and clinical variations of this condition across the two geographical contexts.

## 2. Materials and Methods

This multicenter observational study was conducted over 20 years at referral centers in Santiago de Compostela, Spain, and Mexico City, Mexico, and examined 705 cases of endophthalmitis. Of those, 78 eyes belonged to 67 patients diagnosed with EE: 47 eyes from Spain and 31 eyes from Mexico. Among the total cases studied, EE accounted for 60.25% (47) cases in Spain and 39.74% (31) cases in Mexico. A meticulous review of the medical records of both hospitals identified patients diagnosed with EE between January 2003 and October 2023. EE was clinically diagnosed based on signs of panuveitis with symptoms of decreased visual acuity (VA) with or without ocular pain and suspected concurrent or recent systemic infections. Patients with potentially exogenous causes were excluded. Demographic data and systemic and ocular conditions were assessed. Clinical features, VA at presentation, microbiological findings, treatment modalities, final visual outcomes, and complications were compared between the two cohorts. VA was transformed into logMAR using Snellen and decimal notations [7]. Specifically, counting fingers’ VA was converted to 2.00 logMAR, hand motion (HM) to 2.30 logMAR, and light perception (with or without the ability to perceive projection) to 2.80 logMAR. A complete lack of light perception was recorded in 3.00 logMAR. Various statistical analyses were performed during the univariate analysis, including descriptive statistics, chi-squared tests, and Fisher’s exact tests. Significant variables identified in the univariate analysis were subsequently subjected to multivariate logistic regression. All statistical analyses were performed using SPSS Statistics software (version 22.0; SPSS Inc., Chicago, IL, USA). Statistical significance was defined as *p* < 0.05. This study was approved by the Clinical Research Ethics Committee (Registration 2023/105, RE-24-10).

## 3. Results

### 3.1. Demographic Data

Data from 78 eyes of 67 patients, derived from the Spanish and Mexican cohorts, were meticulously analyzed. The average age of the entire sample was 58 (range, 19–94) years. The Spanish cohort had a mean age of 63 (range, 19–94) years, whereas the Mexican cohort had a mean age of 51 (range, 23–75) years. Although the age spectrum was consistently adult-centric, sex variance was evident. Globally, males accounted for 51.9% of the sample; however, the Spanish sample showed higher male participation (67.39%) compared with the Mexican sample (29.03%). Clinically, the right eye was implicated in 55.13% cases, with bilateral afflictions observed in 27.3% cases (Table 1).

Diabetes mellitus was the dominant risk factor in both groups, with an overall prevalence of 46.8%. Although the Mexican cohort had a higher prevalence (61.3%) than the Spanish cohort (37.0%), the difference was not statistically significant. A notable difference was observed in the use of broad-spectrum antibiotics within the month preceding endophthalmitis diagnosis. The Mexican cohort had a significant prevalence of broad-spectrum antibiotic use in 61.3% cases (*p* = 0.027). Similarly, the past year’s history of hospitalization was also markedly higher in Mexicans, at 61.3% (*p* = 0.016), than in the Spaniards. Urinary tract infections were frequently associated with the Mexican population, with a prevalence of 58.1% (*p* < 0.001). In contrast, advanced-stage neoplasia (28.3%, *p* = 0.013) and remote abscesses, including hepatic abscesses (6.5%), were more prevalent in the Spanish cohort (34.8%, *p* = 0.009) than in the Mexican cohort. Other observed risk factors included positive blood cultures (indicative of bacteremia or fungemia), immunosuppression, end-stage renal failure on dialysis, use of indwelling catheters, endocarditis, meningitis, and dependence. Additionally, toxic habits, such as intravenous drug use, excessive alcohol consumption, and smoking, were prevalent in a substantial portion of the sample (Table 2).

### 3.2. Clinical Features

**Figure 1:**
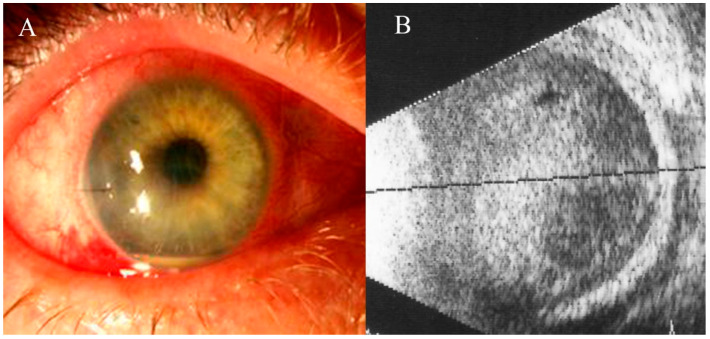
Prevalent signs of EE: (**A**) corneal decompensation and fibrin/hypopyon (**B**), ocular ultrasound scan showing infiltration of the vitreous cavity and retinochoroidal thickening.

The mean initial best-corrected VA was 2.14 ± 0.79 LogMAR, with 35.1% patients having a VA less than HM. The ocular findings in our study included keratic precipitates (26.7%), corneal decompensation (19.5%), fibrin/hypopyon (54.1%), and vitreous haze grade ≥ 3 (74%) (Figure 1).

EE is typically a rapidly evolving disease; however, delays in presentation can occur for various reasons, including neglect, misdiagnosis, or limited access to healthcare services. Additionally, endophthalmitis of fungal origin typically presents in a sub-acute fashion. Therefore, we categorized the cases into early (≤14 days) and delayed (>14 days) presentations to provide a clearer understanding. We found that a notable proportion of cases fell into the delayed presentation category. These delays could be attributed to factors such as lack of awareness about the severity of the condition, initial treatment of systemic symptoms without recognizing the ocular involvement, predominance of fungal etiology, and barriers to accessing specialized ophthalmological care. The duration from the onset of ophthalmological symptoms to referral to the ophthalmology service varied significantly, with a range from 0 to 180 days (mean 13.5 ± 24.65). By presenting these categories, we aimed to highlight the importance of early diagnosis and treatment in improving visual outcomes for patients with EE.

We further analyzed the relationship between the timing of presentation and the improvement in visual acuity (VA) post-treatment. The mean improvement in VA in the early and delayed presentation groups was 0.60 logMAR and 0.46 logMAR, respectively. Although there was a greater degree of improvement in VA in the acute cases, the difference in improvement was not significant (*p* > 0.05).

### 3.3. Causative Organisms and Sources of Infection

Regarding microbiological sampling, 40.3% of the patients had aqueous humor samples collected, 55.2% had vitreous humor samples collected during surgery, and 63.8% had blood cultures. Of those, 14.8%, 54.1%, and 70.0% tested positive for infection, respectively. Notably, the causative organism was accurately identified in 60.3% patients.

Analysis of the prevalence of microorganisms in patient samples from Spain and Mexico revealed significant variations. In the Spanish cohort, bacterial etiology was identified in 57.1% eyes, whereas fungal etiology was predominant in the Mexican cohort in 57.6% eyes. Candida emerged as the predominant microorganism in both countries, accounting for 27.0% cases in Spain and 47.1% cases in Mexico. In Spain, following Candida, the most frequently detected organisms were Staphylococcus (18.9%) and Streptococcus (13.5%). In contrast, following Candida, *Escherichia coli* (*E. coli*) and Staphylococcus were predominant in Mexico, both with a prevalence of 17.6%. Notably, organisms such as *Aspergillus fumigatus* (*A. fumigatus*) and *Klebsiella pneumoniae* (*K. pneumoniae*) had a prevalence of 8.1% in Spanish cases but were not identified in Mexican cases. Conversely, *Actinomyces viscosus*, *Listeria monocytogenes* (*L. monocytogenes*), and Pseudomonas, all of which had a prevalence of 5.9% in Mexico, were absent in Spanish samples. Other microorganisms, such as *Bacteroides caccae*, Brucella, *Enterococcus faecalis*, *Neisseria meningitidis* (*N. meningitidis*), *Serratia liquefaciens*, and Streptomyces, were found only in Spanish samples. These findings underscore the epidemiological differences and similarities between the two countries with respect to the detected microorganisms (Table 3).

The source of infection remained unidentified in 15.8% of the cases. Urinary tract infections were the most prevalent, accounting for 22.4% of the cases, followed by endocarditis, which was observed in 10% of the cases. Subsequently, abdominal origin infections accounted for 7.9% of the cases, and bacteremia due to catheter use accounted for 6.6% of the cases. Among urinary infections, the predominant pathogen was *Candida albicans*, with a frequency of 23.5%, followed by *E. coli* at 17.6%. In cases of endocarditis, the pathogens *K. pneumoniae* and Staphylococcus were notably prevalent, accounting for 28.6% of the cases. *Candida albicans* accounted for one-third (33.3%) of the infections originating from the abdomen, whereas Staphylococcus aureus was detected in a substantial majority of cases related to catheters, comprising 66.7% of the cases (Table S1).

The prevalence of antibiotic resistance was evaluated in 29 different patients, with a comparative focus between Spain and Mexico. In Spain, 28.6% patients reported resistance to the studied antibiotics, whereas 62.5% patients in Mexico exhibited resistance to antibiotics.

In the Spanish cohort, Klebsiella demonstrated 100% resistance to ampicillin and cefuroxime. Additionally, Staphylococcus displayed high levels of resistance to key antibiotics, such as amoxicillin, azithromycin, and clarithromycin, with a particular emphasis on resistance to erythromycin, which was observed in most cultures.

In contrast, a specific portion of the Staphylococcus cultures analyzed in Mexico exhibited resistance to gentamicin. Moreover, noticeable resistance to ceftazidime and clindamycin was observed. Resistance to ciprofloxacin and levofloxacin was also identified in these cultures, along with resistance to fourth-generation quinolones; the latter was not detected in samples from Spain. Additionally, resistance of the bacterium *L. monocytogenes* to vancomycin was recorded, which is particularly concerning, as this antibiotic is often a crucial line of defense. Finally, cases of tobramycin-resistant Pseudomonas and ceftazidime-resistant *K. pneumoniae* were also detected (Table S1).

### 3.4. Treatment Modalities

Medical treatments included topical (90.9%), intraocular (67.0%), and systemic antibiotics (administered intravenously or orally) (98.7%). In all cases, the patients were initially treated with antibiotics intravenously, orally, or intravitreally. Regarding intravitreal drug injections, 67% patients received at least one injection, 47% received at least two injections, and 21% received three or more injections. The most frequently used antibiotics for intravitreal injections were vancomycin (50.88%), ceftazidime (43.86%), and voriconazole (42.98%). Broad-spectrum systemic antibiotics were predominantly administered in most patients.

Vitrectomy was performed in 61.5% eyes, with 27.5% of them requiring a second vitrectomy. Evisceration was performed in 15.4% eyes, and this procedure was more frequently performed in eyes infected with *K. pneumoniae*, *E. coli*, Streptomyces, and *A. fumigatus*.

The average duration of hospitalization was 21 (range, 0–92) days. In Spain, only 6.5% patients were treated as outpatients, whereas in Mexico, 19.4% patients did not require hospitalization.

### 3.5. Early Outcomes and Complications

**Figure 2:**
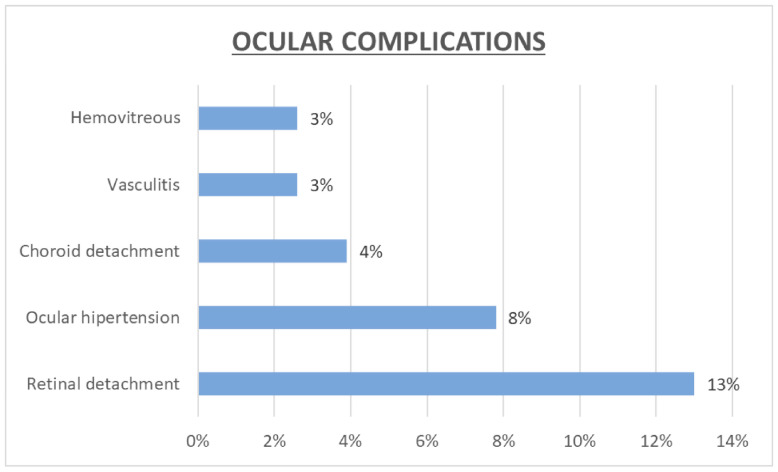
Prevalence of ocular complications in the overall sample.

Various ocular complications were observed following treatment. The most frequently reported complication was retinal detachment or tears, affecting 13.0% of the treated individuals. An increase in intraocular pressure was noted in 7.8% of patients, while hemovitreous was observed in 2.6% of patients. Similarly, vasculitis occurred in 2.6% of patients. Choroidal detachment was slightly more prevalent, affecting 3.9% of patients, whereas panophthalmitis occurred in 10% of patients (Figure 2).

Evisceration was performed in seven instances, accounting for 9.0% of the cases. The most commonly identified pathogens were *E. coli*, which was present in two patients, and *K. pneumoniae*, which was also detected in two patients.

In terms of systemic complications, sepsis was observed in 48% of the cohort, and 41% of the cohort exhibited deterioration in their general health status. Sepsis is defined as a life-threatening organ dysfunction caused by a dysregulated host response to infection [8].

**Figure 3:**
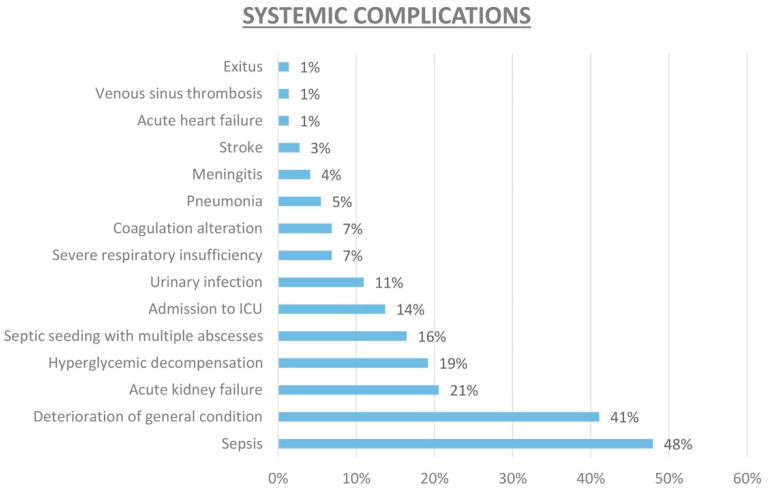
Prevalence of systemic complications in the overall sample.

Additionally, acute renal failure was reported in 21% of patients, and hyperglycemic decompensation occurred in 19% of patients. Among the 67 patients with EE, there were three cases of mortality (representing approximately 4.4% of the total) before completion of the 1 month follow-up (Figure 3).

### 3.6. Visual Prognostic Factors

A significant improvement in post-treatment VA was observed (*p* = 0.003). Functional success was operationally defined as VA of 1.3 logMAR or better, a threshold met by 43.9% of the cases analyzed. No significant differences were observed in relation to preexisting risk factors.

Conversely, the least encouraging outcomes were observed in patients with advanced neoplasia, achieving only an 18.2% functional success rate, and in those with immunosuppression (29.4%). When assessing the functional success of the involved microorganisms, considering those detected in two or more cultures, *Staphylococcus* spp. achieved a functional success rate of 57.1%, whereas *Candida* spp. succeeded in 45.5% of the patients. The most unfavorable outcomes were associated with infections by *Aspergillus* spp., with a success rate of 33.3%, and *Streptococcus* spp., with a success rate of 25%.

No correlation was observed between pre-treatment and post-treatment VA (*p* = 0.203). Furthermore, the analysis revealed no significant differences in final VA outcomes between patients who underwent vitrectomy and those who did not (*p* = 0.738).

## 4. Discussion

In this study, we evaluated EE, a clinically significant ocular infection, by examining cases from Spain and Mexico. Our aim was to identify and compare the risk factors, infection sources, and pathogen patterns between the two populations. The most notable findings were the prevalence of diabetes mellitus, variations in microbial etiology, and differences in sources of infection. These results shed light on the multifaceted nature of EE and provide crucial insights for clinical management and prevention strategies in different geographical settings.

### 4.1. Risk Factors and Foci

Comparing our findings with the global literature, we noted that the prevalence of diabetes mellitus in our cohort (46.8% overall, 61.3% in Mexico, and 37.0% in Spain) aligns with the ranges reported in international studies (14.2–70.7%), where diabetes is identified as a predisposing condition [4,6,7,8,9]. Furthermore, we observed geographical variability in risk factors. For instance, although intravenous drug abuse (IVDA) and hepatobiliary abscesses were prominent in Western and Southeast Asian studies, respectively, they were not predominant in our cohort [4]. This observation underscores the influence of geographical and cultural factors on the prevalence and types of risk factors for EE.

In our study, beyond the prevalence of diabetes mellitus and urinary tract infections, we identified other crucial risk factors that significantly varied between the Spanish and Mexican cohorts. We observed a notable history of recent hospitalization and the predominant use of broad-spectrum antibiotics in the Mexican cohort. This finding suggests a potential association between recent medical care and the development of EE in this population, possibly reflecting the widespread use of intensive medical treatments or greater exposure to hospital environments. Conversely, in the Spanish sample, remote abscesses, immunosuppression, and malignancy were prevalent risk factors. This pattern indicates that, in Spain, conditions that predispose patients to immunosuppression, whether due to chronic diseases or medical treatments, may play a more significant role in EE development.

With respect to specific foci, our study highlights how urinary tract infections and endocarditis influence the incidence of EE. The high prevalence of urinary infections in Mexico (58.1%) suggests a significant infection route in this region. Moreover, we observed that specific pathogens, such as *K. pneumoniae*, which are often associated with hepatic abscesses, appeared to be associated with diabetes, consistent with the trends observed in the Asian population [4,10].

### 4.2. Variance in Microbiological Profiles

Our study demonstrated a clear disparity in the causative microorganisms of EE between the Spanish and Mexican cohorts. Although Candida emerged as the predominant microorganism in both countries, the presence of distinct secondary microorganisms highlights local epidemiological differences. For example, Gram-positive bacteria and certain fungi were isolated more frequently from the Spanish cohort, whereas the presence of species, such as *E. coli* and Staphylococcus, was notable in the Mexican cohort.

Our findings support the notion that geographical location significantly influences microbiological profiles. In East Asia, Gram-negative bacteria, especially *K. pneumoniae*, predominate [10,11]. Historically, *K. pneumoniae* infections were primarily hospital-acquired, affecting individuals with compromised immune systems. However, a novel hypervirulent strain has been identified that commonly causes community-acquired infections, notably presenting as pyogenic liver abscesses, which is a global health concern. This strain is particularly prevalent in East Asia, with a significant number of cases reported in Taiwan, South Korea, and China. The hypervirulence of this strain is largely attributed to specific capsular serotypes, especially K1 and K2, which produce a robust mucoviscous polysaccharide capsule that resists phagocytosis and enhances pathogenicity. Individuals with diabetes mellitus are at a higher risk of infection, as poor glycemic control impairs neutrophilic phagocytosis, facilitating bacterial proliferation in the liver [10,12].

In contrast, in North America and Australia, fungemia, often associated with IVDA and the presence of intravenous catheters, leads to the frequent isolation of fungi and Gram-positive cocci, with Candida species commonly identified [4,6]. This global variability underscores the complexity of EE infection patterns and the influence of regional factors.

### 4.3. Management and Resistance

Although our study showed a high use of topical, systemic, and intraocular antibiotics, the literature indicates no specific treatment guidelines for EE. Vitrectomy was performed in 61.5% cases in our study, which is in the higher range reported in the literature, where the rates vary from 14% to 66% for EE in general [3,12]. The fact that 27.5% patients in our study required a second vitrectomy highlights the complexity of managing EE. However, the repetition of vitreous surgery various in existing studies, depending on the nature of the infection [13,14].

In our study, aqueous humor samples were obtained from 40.3% patients, vitreous humor samples from 55.2% patients, and blood cultures from 39.0% patients, which had infection positivity rates of 14.8%, 54.1%, and 70.0%, respectively. These results are consistent with the variability reported in other studies, where blood culture positivity rates for infection ranged from 0 to 100% [15] and vitreous humor culture rates ranged from 22 to 100% [11,16].

Our study revealed high levels of antibiotic resistance in Staphylococcus. Notably, over a quarter of the Staphylococcus samples showed resistance to quinolones, including fourth-generation quinolones in some cases. Therefore, the initial choice of antibiotics should be based on general knowledge of the resistance patterns of different species along with adjustment according to laboratory results for antibiotic sensitivity and clinical progression. Moreover, early identification of diagnostic parameters and risk factors can influence diagnostic accuracy, and treatment options can be tailored based on the severity and progression of the disease, along with the identification of specific pathogens, improving patient outcomes. The clinical assessments and treatment pathways based on individual findings are illustrated in Table S2. However, further research is required to provide clinicians with a clear framework for managing EE, enhancing both diagnostic accuracy and treatment efficacy.

### 4.4. Outcomes and Complications in Endogenous Endophthalmitis

The results of our study highlight a significant improvement in post-treatment VA in patients with endophthalmitis. We observed functional success, defined as a VA of 1.30 logMAR or better, in 43.9% of the cases analyzed. Comparing these results with the available data, our study revealed a notable variability in VA outcomes. In cases of EE caused by fungi, we achieved a functional success rate of 41.7%, whereas in cases caused by bacteria, the success rate was 52.4%. Connell et al. found a VA ≥ 20/400 in 35.4% bacterial cases and 74% fungal cases [6]. Additionally, more recent studies have indicated that between 26 and 75% of eyes achieve a VA greater than counting fingers [13,17]. Additionally, in our study, we observed that 9% of the patients required evisceration and 4.5% succumbed to their conditions. This outcome is consistent with those of other studies in the field, where mortality rates in similar cases have been reported to vary between 4% and 21.1% [1,12].

### 4.5. Limitations and Future Research Directions

This study has some limitations. First, the retrospective design inherently carries the risk of bias, as it depends on historical records that may be outdated or incomplete. This can lead to potential inaccuracies in the collected data and affect the reliability of the findings. Second, the data for this study were gathered from multiple sources, especially in Mexico. The Eye Hospital in Mexico City does not have an internal and infectious medicine service, necessitating referrals to tertiary hospitals for comprehensive patient care. Thus, the multisource data collection could have introduced inconsistencies and variability in the dataset, potentially impacting the overall conclusions. Furthermore, the reliance on medical records that are up to two decades old is a limitation, as changes in diagnostic criteria, treatment protocols, and recording practices over time could distort the statistical analyses. Therefore, these factors must be considered when interpreting the study results, despite efforts to ensure data accuracy. Notwithstanding these limitations, this study provides valuable insights into the epidemiological and clinical aspects of EE in diverse geographical settings.

To address the constraints associated with retrospective data, future research could benefit from employing a prospective design, which would allow for real-time data collection, minimizing the biases and inaccuracies inherent in historical records. Additionally, enforcing uniform data-gathering techniques across different centers could significantly enhance the consistency and reliability of the data. Moreover, including a broader range of geographical regions in future studies would provide a more thorough comprehension of EE. This would help with identifying region-specific risk factors, microbial patterns, and treatment outcomes, thereby enabling the development of tailored clinical management strategies. Furthermore, conducting a comprehensive examination of subgroups categorized by specific bacteria and patient demographics could yield more profound insights. Finally, understanding the interactions between different pathogens and host factors such as age, sex, and comorbidities would facilitate more personalized and effective treatment approaches. Thus, we can advance our understanding of EE and improve patient care across diverse populations by addressing these aspects in future research.

## 5. Conclusions

In conclusion, this study highlights the effects of geographical and cultural factors on EE. Variations in risk factors, infection sources, and pathogen profiles between Spanish and Mexican cohorts underscore the need for tailored clinical management and prevention strategies. Therefore, healthcare providers should consider local nuances, such as the prevalence of diabetes or specific pathogens, when addressing EE. Public health initiatives must adapt to regional factors by implementing targeted education, improving healthcare access, and developing region-specific guidelines. A holistic approach that recognizes these influences can enhance EE prevention, early detection, and treatment, ultimately benefiting patients and reducing the burden of ocular infections.

## Figures and Tables

**Table 1:** Demographic characteristics in the Spanish and Mexican cohorts.

Sample Characteristics	Global	Spain	Mexico
Mean age (range)	58	63	51
	(19–94)	(19–94)	(23–75)
Sex (men)	51.90%	67.39% *	29.03% *
Laterality (right eye)	55.13%	53.19%	58.06%
Bilaterality	27.30%	19.57%	38.71%
Rural background	54.50%	73.91% *	25.81% *
Recent hospitalization	43.80%	28.30%	61.30%

An asterisk (*) indicates *p* < 0.05.

**Table 2:** Prevalence of systemic risk factors in the Spanish and Mexican cohorts.

Risk Factor		Spain		Mexico		*p*
Advanced-stage neoplasia						
	No	33	65.2%	29	93.5%	0.013
	Yes	13	28.3%	1	3.2%	
Immunosuppression						
	No	28	60.90%	24	77.40%	0.261
	Yes	15	32.60%	5	16.10%	
Diabetes mellitus						
	No	26	56.50%	11	35.50%	0.109
	Yes	17	37.00%	19	61.30%	
Endocarditis						
	No	35	76.10%	30	96.80%	0.035
	Yes	8	17.40%	0	0.00%	
Intravenous drug abuse (IVDA)						
	No	37	80.40%	29	93.50%	0.256
	Yes	6	13.00%	1	3.20%	
Dependence						
	No	40	87.00%	28	90.30%	0.814
	Yes	3	6.50%	2	6.50%	
Meningitis						
	No	39	84.80%	30	96.80%	0.155
	Yes	5	10.90%	0	0.00%	
Urinary tract infection						
	No	35	76.10%	12	38.70%	<0.001
	Yes	8	17.40%	18	58.10%	
Recent use of broad-spectrum systemic antibiotics (on the last month)						
	No	29	63.00%	11	35.50%	0.027
	Yes	14	30.40%	19	61.30%	
Recent hospitalization (at least 15 days within the last year)						
	No	30	65.20%	11	35.50%	0.016
	Yes	13	28.30%	19	61.30%	
Parenteral nutrition						
	No	43	93.50%	30	96.80%	0.523
Remote abscess						
	No	27	58.70%	28	90.30%	0.009
	Yes	16	34.80%	2	6.50%	
Indwelling catheter carrier						
	No	39	84.80%	27	87.10%	0.811
	Yes	4	8.70%	3	9.70%	
Positive blood cultures (sepsis, bacteremia, or fungemia)						
	No	22	47.80%	21	67.70%	0.223
	Yes	22	47.80%	9	29.00%	

**Table 3:** Microorganisms identified in Spanish and Mexican ocular and systemic samples.

Microorganisms		Spain	Mexico
*Fungi*	Candida	27.0%	47.1%
	*Aspergillus fumigatus*	8.1%	0.0%
*Gram-negative bacteria*	Brucella	2.7%	0.0%
	*Neisseria meningitidis*	2.7%	0.0%
	*Bacteroides caccae*	2.7%	0.0%
	*Escherichia coli*	5.4%	17.6%
	*Klebsiella pneumoniae*	8.1%	0.0%
	Pseudomonas	0.0%	5.9%
	*Serratia liquefaciens*	2.7%	0.0%
*Gram-positive bacteria*	Staphylococcus	18.9%	17.6%
	Streptococcus	13.5%	0.0%
	Streptomyces	5.4%	0.0%
	*Actinomyces viscosus*	0.0%	5.9%
	*Listeria monocytogenes*	0.0%	5.9%
	*Enterococcus faecalis*	2.7%	0.0%

## Data Availability

Data is contained within the article/Supplementary Material.

## Supplementary Materials

The following supporting information can be downloaded at https://www.mdpi.com/article/10.3390/jcm13174990/s1, Table S1: Prevalence of the different microorganisms involved depending on the systemic risk factors exhibited. Table S2: Flowchart outlining the clinical assessment and treatment processes for EE.
