# Supplementary material for: Two-Decade Retrospective Analysis of Endogenous Endophthalmitis in Spain and Mexico: A Comprehensive Study
Source: J Clin Med. 2024 Aug 23;13(17):4990. doi: 10.3390/jcm13174990 (PMC11396706; doi:10.3390/jcm13174990)
Supplement: Supplementary file 1 [file jcm-13-04990-s001.zip › jcm-3119619-supplementary.pdf]

		Aspergillus fumigatus		*Bacteroides caccae*		*Brucella*		*Candida*		*Escherichia coli*		*Klebsiella pneumoniae*		*Listeria monocytogenes*		*Neisseria meningitidis*		*Serratia liquefaciens*		*Staphylococcus*		*Streptococcus*	
		n	%	n	%	n	%	n	%	n	%	n	%	n	%	n	%	n	%	n	%	n	%
Advanced-stage neoplasia	No	2	5.9	0	0	1	2.9	12	35.3	3	8.8	3	8.8	1	2.9	1	2.9	1	2.9	8	23.5	2	5.9
	Yes	1	9.1	1	9.1	0	0	4	36.4	1	9.1	0	0	0	0	0	0	0	0	2	18.2	2	18.2
Immuno suppression	No	0	0	0	0	1	3.6	10	35.7	2	7.1	3	10.7	0	0	1	3.6	1	3.6	8	28.6	2	7.1
	Yes	3	18.8	1	6.3	0	0	5	31.3	2	12.5	0	0	1	6.3	0	0	0	0	2	12.5	2	12.5
DM	No	3	11.1	1	3.7	1	3.7	11	40.7	2	7.4	0	0	0	0	1	3.7	1	3.7	3	11.1	4	14.8
	Yes	0	0	0	0	0	0	5	27.8	2	11.1	3	16.7	1	5.6	0	0	0	0	7	38.,9	0	0
Liver abscess	No	3	7.1	1	2.4	1	2.4	16	38.1	4	9.5	0	0	1	2.4	1	2.4	1	2.4	10	23.8	4	9.5
	Yes	0	0	0	0	0	0	0	0	0	0	3	100	0	0	0	0	0	0	0	0	0	0
IVDA	No	3	7.9	1	2.6	0	0	10	26.3	4	10.5	3	7.9	1	2.6	1	2.6	1	2.,6	10	26.3	4	10.5
	Yes	0	0	0	0	1	14.3	6	85.7	0	0	0	0	0	0	0	0	0	0	0	0	0	0
Urinary tract infection	No	3	10.7	1	3.6	1	3.6	12	42.9	1	3.6	0	0	0	0	1	3.6	1	3.6	4	14.3	4	14.3
	Yes	0	0	0	0	0	0	4	23.5	3	17.6	3	17.6	1	5.9	0	0	0	0	6	35.3	0	0
Recent hospitalization	No	1	3.4	0	0	0	0	9	31	3	10.3	3	10.3	0	0	1	3.4	1	3.4	7	24.1	4	13.8
	Yes	2	12.5	1	6.3	1	6.3	7	43.8	1	6.3	0	0	1	6.3	0	0	0	0	3	18.8	0	0
Remote abscess	No	2	6.3	1	3.1	1	3.1	13	40.6	4	12.5	0	0	0	0	1	3.1	1	3.1	6	18.8	3	9.4
	Yes	1	7.7	0	0	0	0	3	23.1	0	0	3	23.1	1	7.7	0	0	0	0	4	30.8	1	7.7
Endocarditis	No	3	7.9	1	2.6	1	2.6	16	42.1	3	7.9	1	2.6	1	2.6	1	2.6	1	2.6	8	21.1	2	5.3
	Yes	0	0	0	0	0	0	0	0	1	14.3	2	28.6	0	0	0	0	0	0	2	28.6	2	28.6
Indwelling catheter	No	3	7.7	1	2.6	1	2.6	14	35.9	4	10.3	3	7.7	1	2.6	1	2.6	1	2.6	6	15.4	4	10.3
	Yes	0	0	0	0	0	0	2	33.3	0	0	0	0	0	0	0	0	0	0	4	66.7	0	0
Sepsis	No	0	0	1	4.5	0	0	12	54.5	1	4.5	1	4.5	1	4.5	1	4.5	1	4.5	3	13.6	1	4.5
	Yes	3	12.5	0	0	1	4.2	4	16.7	3	12.5	2	8.3	0	0	0	0	0	0	7	29.2	4	16.7

Table S1. *Prevalence of the different microorganisms involved depending on the systemic risk factors exhibited.*

INITIAL ASSESSMENT

__Symptoms:__ Decreased visual acuity, ocular pain
__Signs:__ Vitreous haze +/- anterior chamber inflammation, corneal edema, conjunctival hyperemia
__Risk Factors:__ Diabetes mellitus, recent hospitalization, recent broad-spectrum antibiotic use, malignancy, immunosuppression, endocarditis, intravenous drug abuse, urinary tract infection, parenteral nutrition, remote abscesses, indwelling catheter, etc.

DIAGNOSTIC PARAMETERS

__Microbiological exam:__ Aqueous and vitreous humor samples +/- blood cultures
__Imaging:__ Ocular ultrasound
Systemic guided assessment to identify focus of infection

MICROBIAL IDENTIFICATION

Always initiate empirical broad-spectrum antibiotics
__Positive Culture Results:__ Specific antibiotic or antifungal treatment
__Negative Culture Results:__ Empirical broad-spectrum antibiotics based on clinical response

TREATMENT PATHWAYS

__Mild Disease:__

- ◊ <u>Antibiotics/antifungals</u>: topical, intravitreal and systemic

Evaluate the need of:

- ◊ <u>Corticosteroids</u>: local/systemic

__Moderate Disease:__

- ◊ <u>Antibiotics/antifungals</u>: topical, intravitreal and systemic

Evaluate the need of:

- ◊ <u>Corticosteroids</u>: local/systemic
- ◊ Repeating <u>intravitreal antibiotic/antifungal injections</u>
- ◊ <u>Vitrectomy</u>
- ◊ <u>Adjuvant treatments</u>

__Severe Disease:__

- ◊ <u>Antibiotics/antifungals</u>: topical, intravitreal and systemic
- ◊ <u>Corticosteroids</u>: local/systemic
- ◊ <u>Vitrectomy</u>

Evaluate the need of:

- ◊ Repeating <u>intravitreal antibiotic/antifungal injections</u>
- ◊ Repeating <u>vitrectomy</u>
- ◊ <u>Adjuvant treatments</u>
- ◊ <u>Evisceration</u> in refractory cases

|

FOLLOW-UP

|

Regular monitoring of ***visual acuity***<br>Repeat ***cultures*** if initial treatment fails<br>***Adjust treatment*** based on clinical response and culture results

Table S2: Flowchart outlining the clinical assessment and treatment processes for EE.
